# Live intraocular *Angiostrongylus cantonensis* infection in Vietnam: a case report

**DOI:** 10.1016/j.ajoc.2026.102703

**Published:** 2026-10-24

**Authors:** Hai Xuan Ho, Thanh Ngoc Tran, Thoa Thi Doan, Bich Nguyen Ngoc

**Affiliations:** a Department of Vitreous and Retina, Vietnam National Eye Hospital, Hanoi, Viet Nam; b Department of General Ophthalmology, Cung Hong Son International Eye Hospital, Hanoi, Viet Nam; c Department of Parasitology, Hanoi Medical University, Hanoi, Viet Nam; d Hanoi Medical University Hospital, Hanoi, Viet Nam

**Keywords:** *Angiostrongylus cantonensis*, Ocular angiostrongyliasis, Anterior chamber

## Abstract

**Purpose:**

To report a rare case of intraocular angiostrongyliasis caused by *Angiostrongylus cantonensis* in Vietnam and to highlight its clinical features and management.

**Observations:**

A patient presented with a live, motile nematode initially observed in the vitreous cavity, which subsequently migrated into the anterior chamber. The parasite was successfully removed through surgical extraction from the anterior chamber. Morphological examination and molecular analysis confirmed the organism as *Angiostrongylus cantonensis*. Postoperative treatment with antihelminthic medication and corticosteroids resulted in a favorable visual recovery without recurrence of ocular inflammation.

**Conclusions and importance:**

Intraocular involvement of *Angiostrongylus cantonensis* is a rare manifestation of human angiostrongyliasis. This case highlights that early diagnosis and timely surgical removal of the parasite are critical for achieving favorable visual outcomes and preventing serious ocular complications, particularly in endemic regions.

## 1 Introduction

Human angiostrongyliasis is a foodborne helminthic zoonosis predominantly caused by *Angiostrongylus cantonensis* and is widely recognized in endemic regions of Southeast Asia, China, and the Pacific Islands. Humans are accidental hosts that become infected by ingesting snails, slugs, or paratenic hosts containing infective larvae, as well as vegetables contaminated with these larvae.^1^

Within the genus *Angiostrongylus*, *A. cantonensis*, commonly known as the rat lungworm, is one of only two species known to cause human disease and is the primary etiologic agent of eosinophilic meningitis and meningoencephalitis in many countries.^2^ By 2008, approximately 2800 human cases had been reported worldwide.^1^

Ocular angiostrongyliasis represents an uncommon manifestation of the disease, accounting for approximately 1.1% of reported human cases^3^ and may lead to significant visual impairment. Globally, more than 45 ocular cases have been reported across 10 countries, with nearly half originating in Thailand and the remainder in other Asian regions.^4^, ^5^, ^6^, ^7^ In Vietnam, most reported cases have involved eosinophilic meningitis,^8^ whereas ocular involvement has been infrequently described. To date, only two ocular cases have been reported, one in 2002, and another in 2015 that included molecular confirmation.^5^, ^9^ Here, we present an additional case of intraocular angiostrongyliasis caused by *A. cantonensis*.

## 2 Case report

A 70-year-old male farmer from a rural province presented to the Vietnam National Eye Hospital with gradual blurring of vision in his right eye and the perception of moving shadows in the sky, which he described as seeing “a flying dragon” for more than 10 days. He reported no redness, no pain, and no history of trauma or prior interventions to the eye. He had a habit of working in the fields without protective gear and poor hygiene, particularly handwashing before meals. His diet is predominantly non-vegetarian, with a notable preference for boiled golden apple snails, which he eats frequently. He has a history of hypertension that he has not consistently managed.

During his initial examination, the best corrected visual acuity (BCVA) was counting fingers at a distance of 3 m in the right eye and 20/160 in the left eye. A slit lamp examination revealed no significant lesions in the anterior segments of either eye. Bilateral nuclear sclerosis grade III was noted (LOCS III). The fundoscopic examination showed a moving nematode in the inferior half of the vitreous cavity of the right eye, which reacted to the light source. There was minimal vitreous inflammation and mild macular edema present in the right eye. Both eyes showed signs of stage I hypertensive retinopathy, as there was mild narrowing of the small blood vessels. No evidence of chorioretinitis, subretinal migratory tracts, or abnormal optic discs was found. Extraocular movements were normal, and the ocular adnexa area showed no lesions. A systemic exam found no signs of migratory subcutaneous tracks or nodules.

His B-scan showed layered hyperechoic lesions in the posterior vitreous chamber of the right eye, whereas the left eye appeared normal (Fig. 1A). Optical coherence tomography (OCT) revealed mild diffuse macular edema with attenuation of the ellipsoid zone in the right eye (Fig. 1B); the left eye was normal. Fundus photography was not performed due to limited imaging equipment availability at that time.

**Fig. 1:**
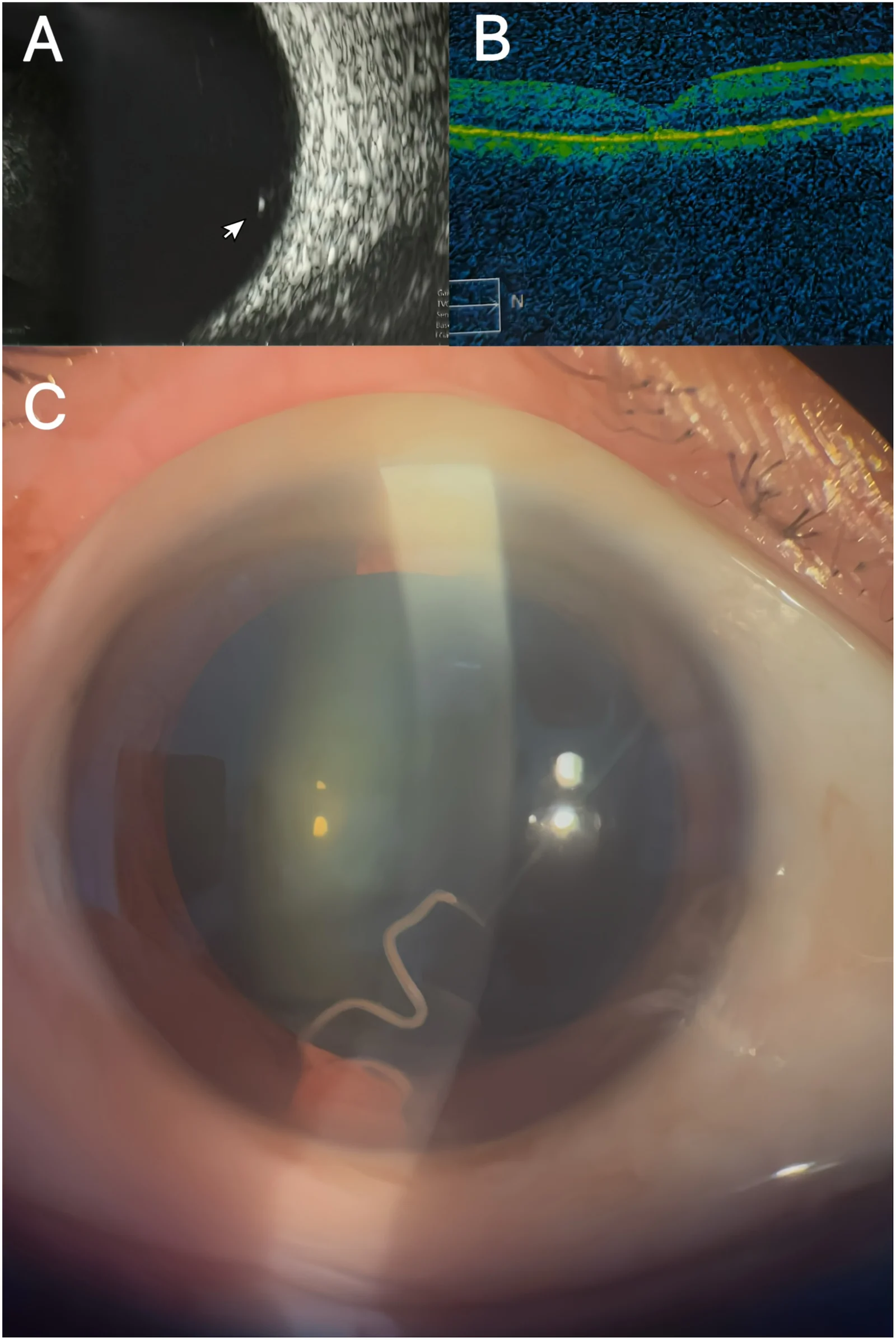
**Initial clinical and imaging findings of the right eye.** (A) B-scan ultrasonography revealed a layered echogenic lesion in the posterior vitreous, adjacent to the retinal surface, suggestive of an intraocular parasite (arrow). Notably, the surrounding vitreous exhibited minimal inflammatory reaction. (B) OCT demonstrated mild macular edema and ellipsoid zone attenuation. (C) One week later, a live nematode was observed freely moving in the anterior chamber.

Systemic tests, including eosinophil count and liver and renal function assessments, were normal. However, the patient presented with elevated blood pressure of 150/70 mmHg, as he had not been taking antihypertensive medication.

The option of pars plana vitrectomy to remove the intraocular parasite was discussed in detail with the patient. Due to financial constraints, he was unable to be admitted to the hospital for surgery. Topical non-steroidal anti-inflammatory eye drops were prescribed to alleviate inflammation, and the patient was advised to optimize blood pressure control and return for surgical treatment as soon as possible.

One week later, he returned to the hospital for surgery, as his BCVA in the right eye had declined to counting fingers at 0.5 m, while the left eye remained unchanged from the previous examination. During the slit-lamp examination of the right eye, a freely moving nematode was observed in the lower half of the anterior chamber (AC), with no attachment to any surrounding structures (Fig. 1C). Minimal conjunctival congestion and corneal edema were noted, along with defects in Zinn's zonules at the 6 to 7 o'clock position. Fundoscopic examination revealed no other parasites in the vitreous, only mild vitreous opacities. Left eye examination continued to show no change since the last visit.

The patient was admitted for urgent surgical removal to prevent the parasite from migrating back into the vitreous cavity. The procedure was performed under topical anesthesia. We performed a single-plane incision of approximately 2 mm at the 11 o'clock position using a 15-degree blade. Intracameral injection of 0.3 mL preservative-free 1% lidocaine hydrochloride was administered to immobilize the nematode. We then slowly injected dispersive viscoelastic agents (2% hydroxypropyl methylcellulose) into the AC to coat the endothelium, maintain space, and reduce damage to surrounding structures. As the AC filled with the viscoelastic substance, the nematode became embedded within it. Visco-expression was subsequently performed by slowly injecting viscoelastic from a site opposite the incision while applying gentle external pressure at the wound edge. This created a controlled flow that allowed the nematode to be delivered through the incision. Care was taken to avoid direct instrument contact with intraocular structures and to ensure the parasite remained intact for later identification. Preserving the integrity of the worm was considered important to reduce the risk of postoperative inflammatory reaction.

Once the nematode was removed (Fig. 2), the viscoelastic was cleared and replaced with balanced salt solution to reform the chamber. The live nematode was sent to the parasite lab for identification.

**Fig. 2:**
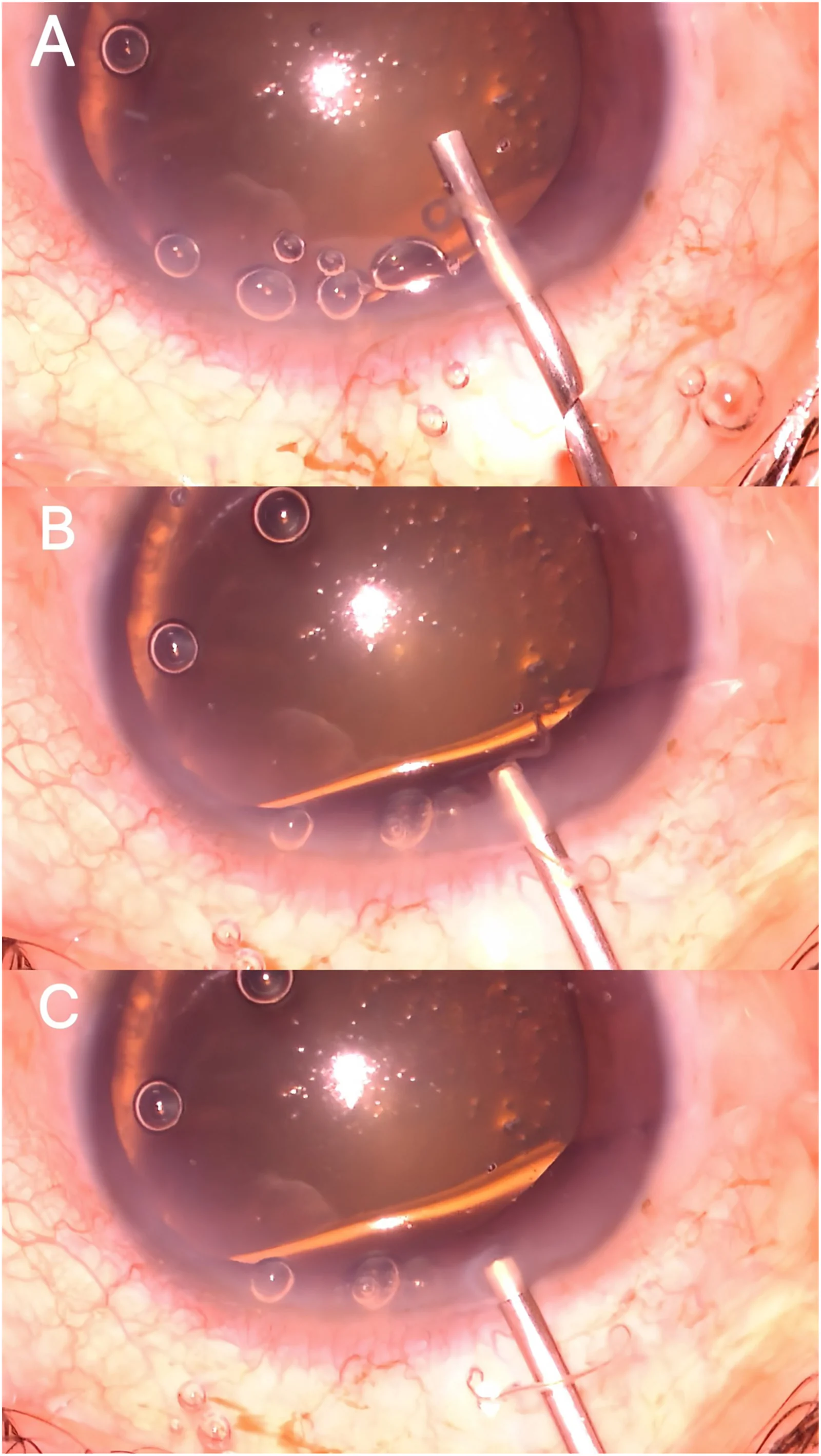
**Surgical removal of the live nematode from the anterior chamber.** (A) The parasite was immobilized with intracameral lidocaine and embedded in viscoelastic material. (B) Partial extrusion of the worm through the surgical incision during visco-expression. (C) Complete removal of the intact nematode.

Postoperatively, moxifloxacin 0.5% and dexamethasone 0.1% eye drops were prescribed to be instilled four times daily, along with oral albendazole 400 mg twice daily for 1 week. Five days after surgery, his BCVA in the right eye improved to counting fingers at 2.5 m. Slit lamp examination showed mild conjunctival congestion, corneal edema, and flare (1+) in the AC. Multiple white fleck-like lesions and indented lesions were noted in the center of the anterior capsule, but there were no signs of a progressive cataract. Therefore, we decided to postpone cataract surgery and monitor the lesions.

During the two-week follow-up, the patient's right eye BCVA improved significantly to 20/160. The slit lamp exam showed improved conjunctival congestion, resolved corneal edema and flare in the AC. The anterior capsule lesions appeared “hardened” with contracted, indented areas (Fig. 3A). Fundoscopy revealed no inflammation, but rather mild vitreous opacities. OCT revealed resolution of macular edema and partial recovery of the ellipsoid zone (Fig. 3B).

**Fig. 3:**
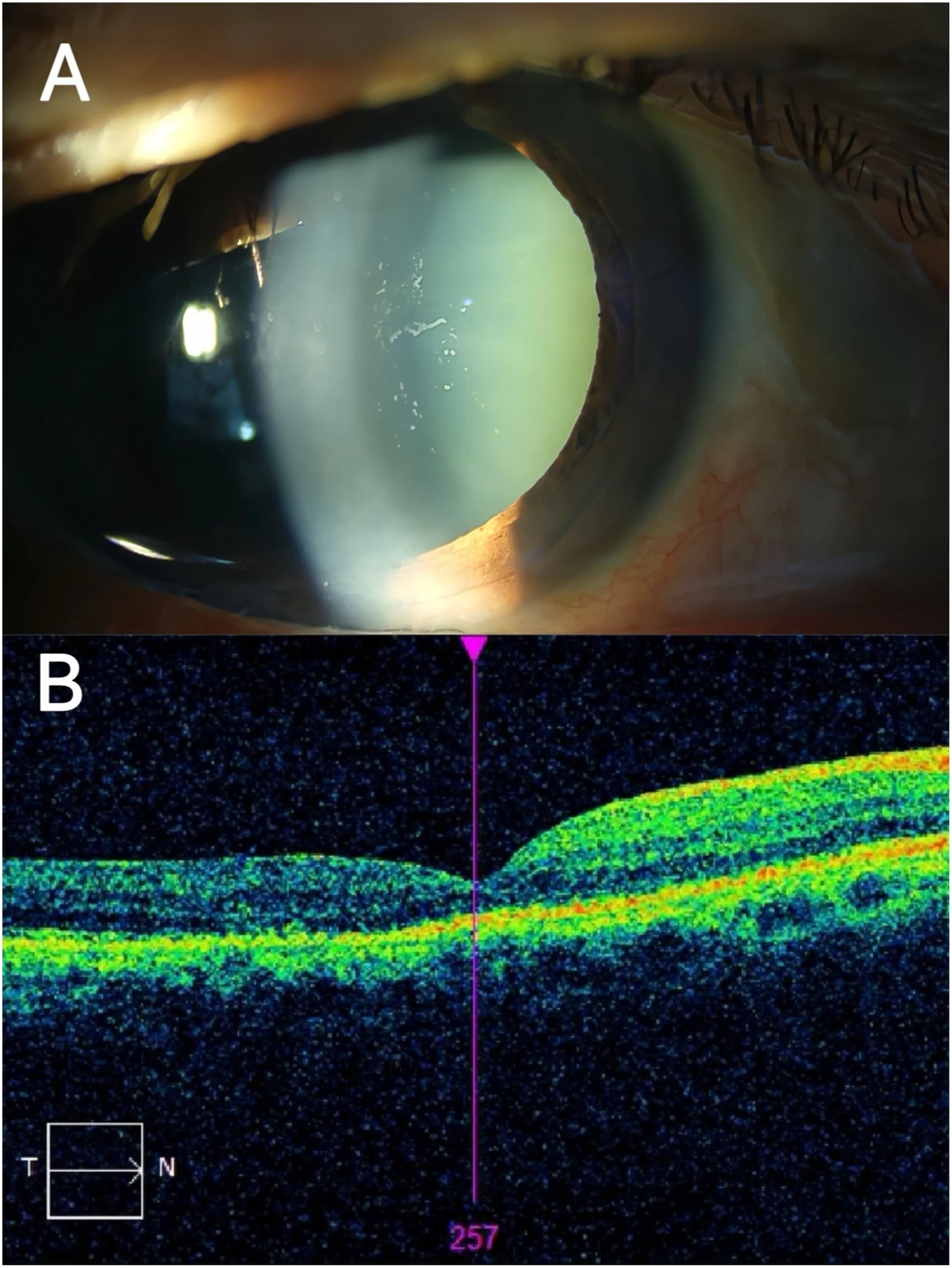
**Postoperative findings at 2 weeks.** (A) Slit-lamp photograph showing stable whitish fleck-like lesions on the anterior lens capsule with adjacent indentation areas. (B) OCT demonstrating reduction of macular edema and partial recovery of the ellipsoid zone.

Due to financial and geographic constraints, follow-up care was limited. The patient was educated on personal hygiene, avoiding undercooked snails to prevent reinfection, and managing blood pressure. His recovery remains stable at 3 months post-operation, as he has reported back to us despite not being able to attend follow-ups.

The extracted worm was examined by a parasitologist. Gross examination revealed a thin, translucent, convoluted nematode measuring approximately 2.0 cm in length and 70-80 μm in width. Microscopic evaluation showed a slender, cylindrical body that tapered toward both ends. The anterior end was rounded and measured 30-40 μm in breadth, with a simple oral opening lacking a buccal capsule that opened directly into a muscular esophagus. A long, tubular intestine arranged in a spiral configuration was observed. The posterior end was blunt, approximately 40 μm wide, and the cuticle was smooth without kinks or surface projections. These morphological characteristics were consistent with *A. cantonensis* (Fig. 4). Subsequent molecular analysis with BLAST comparison against the GenBank database demonstrated high sequence similarity to *A. cantonensis* (GenBank accession no. PP729488.1), thereby confirming the species identity.

**Fig. 4:**
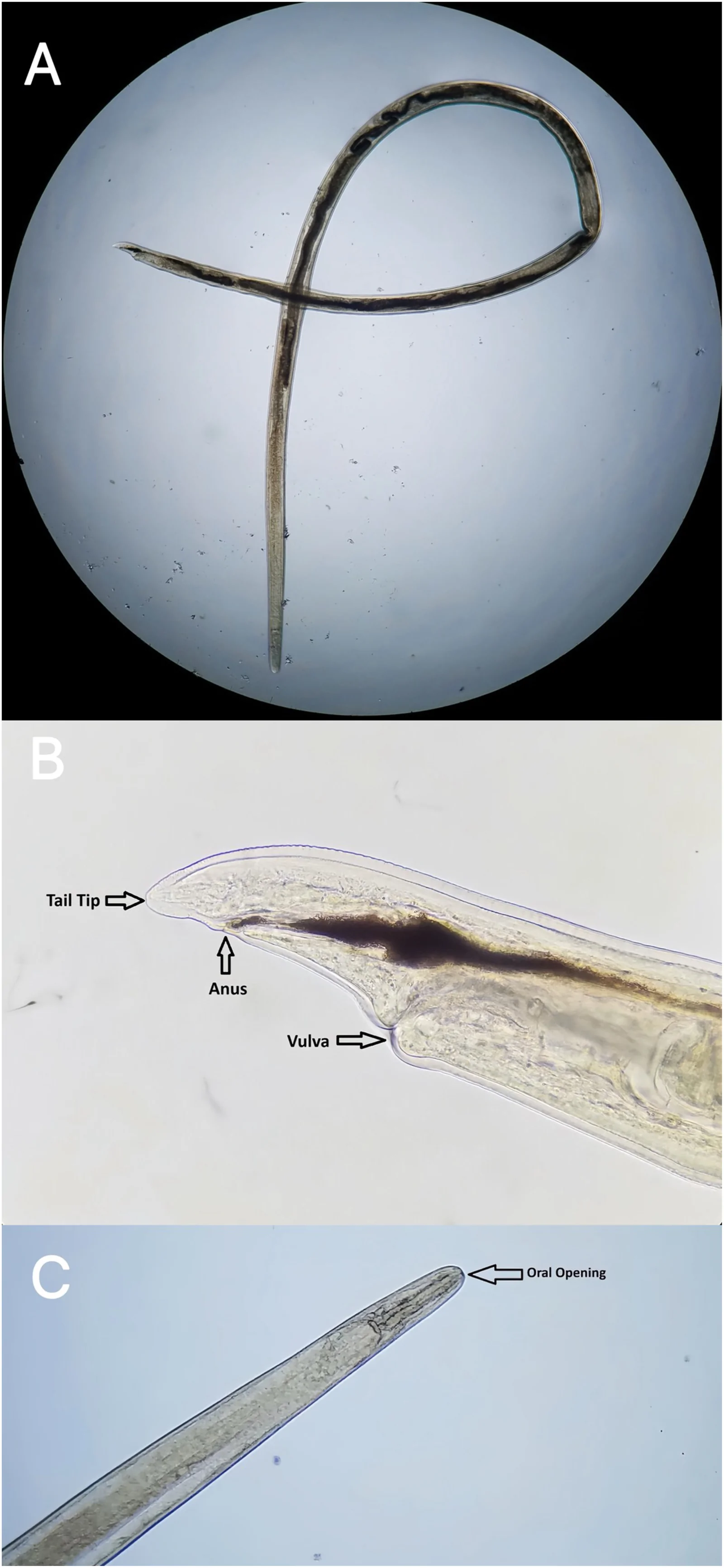
**Morphologic features of the extracted nematode.** (A) Whole specimen demonstrating a slender, translucent body. (B) Posterior end showing the tail tip, anus and vulvar opening. (C) Anterior end displaying the oral opening.

## 3 Discussion

Human angiostrongyliasis is a foodborne zoonosis primarily characterized by eosinophilic meningitis, although ocular involvement is a rare manifestation. In Vietnam, only two cases of ocular angiostrongyliasis have been reported to date. The first case involved a 3-year-old girl with a history of consuming raw snails, who presented with a high fever and a red, painful right eye. Upon examination, an actively mobile worm was discovered in the AC and was successfully surgically removed and morphologically identified.^9^ Our case shares a similar history of consuming snails, particularly golden apple snails, which are well-known intermediate hosts of *A. cantonensis* and are commonly used in traditional Vietnamese cuisine. However, unlike the first case, our patient did not exhibit systemic symptoms, and the AC inflammation was minimal. This difference may be attributed to the age-related inflammatory response to the parasite, which tends to be more intense in younger individuals and decreases with age.^10^

The other case reported in Vietnam involved a 23-year-old female who experienced dimness in her vision and pain due to a bulge in her left eye.^5^ Although not classified as an intraocular case, the report included molecular identification of the parasite. Molecular techniques can provide more precise information regarding the causative agent, as differentiation between *Angiostrongylus* species can be challenging due to overlapping size descriptions and morphological similarities.^11^ In our case, molecular analysis was also performed to support definitive species identification and to assist in clinical assessment and epidemiologic understanding.

Worldwide reports indicate that ocular angiostrongyliasis most commonly involves the AC or vitreous cavity, whereas subretinal localization is less common. Posterior segment cases may have poorer outcomes even after successful worm removal or laser treatment. Reported sequelae include retinal pigment epithelial changes, a subretinal white net, chorioretinal atrophy, and recurrent retinal detachment leading to profound vision loss in severe cases.^12^, ^13^, ^14^ These changes may result from local chorioretinal inflammation, host immune responses, or direct tissue injury caused by the parasite. In our case, the live worm migrated from the vitreous cavity to the AC. This migration made surgical removal less challenging and may have reduced further injury to the posterior segment. For AC removal, experience from the literature suggests that using 0.3 mL of 1% preservative-free lidocaine intracamerally can effectively immobilize motile parasites and, when combined with viscoelastic agents, allow for the safe removal of the parasite while protecting ocular structures.^15^, ^16^ After immobilizing the parasite and embedding it in viscoelastic agents, some authors attempted to retrieve the nematode from the AC using forceps or a Simcoe cannula.^7^, ^17^ However, we do not recommend this technique, as direct manipulation may apply excessive pressure on the parasite, leading to its demise. Such damage can hinder subsequent identification and may result in the release of antigens, which could trigger a severe hypersensitivity reaction post-surgery.^18^ The visco-express method, as described in the literature for the extraction of cysticercus cysts from the AC,^19^ is preferred as a safer option for complete, no-touch removal of parasites, both in our case and in others.^6^, ^15^, ^20^

In terms of medical therapy, there is limited evidence indicating that oral antihelminthic agents, such as albendazole and ivermectin, can achieve sufficient intraocular levels to effectively eradicate the parasite. Even if these medications reached adequate intraocular levels and eliminated the parasite, the parasite's death could trigger the release of significant amounts of antigens, which may provoke severe inflammation.^18^ For live intraocular parasites, current practice remains surgical removal as the main approach, with systemic anthelmintics used to clear systemic infection rather than to achieve therapeutic concentrations within the eye.

## 4 Conclusion

Ocular angiostrongyliasis is a rare, potentially sight-threatening zoonotic disease caused by the parasite *A. cantonensis*. Timely diagnosis and, when feasible, complete surgical removal of the parasite are the best approaches to prevent blindness in affected patients. Public education on the hazards of consuming undercooked or raw foods is crucial for reducing the risk of this infection.

## CRediT authorship contribution statement

**Hai Xuan Ho:** Writing – review & editing, Methodology, Investigation, Conceptualization. **Thanh Ngoc Tran:** Writing – review & editing, Writing – original draft, Visualization, Conceptualization. **Thoa Thi Doan:** Writing – review & editing, Investigation. **Bich Nguyen Ngoc:** Writing – review & editing, Validation, Methodology, Investigation.

## Ethics declaration

Written informed consent to take part in the study and to publish the article has been obtained from all participants or their legal representatives. The privacy rights of participants have been observed.

This study was performed in compliance with relevant laws, regulatory frameworks and guidelines where the research took place. Ethics committee approval was not required under relevant laws and institutional guidelines. Ethics committee approval was not required for this single-patient case report in accordance with institutional policy.

This research follows the CARE guidelines and the CARE checklist.

## Patient consent

Written informed consent was obtained from the patient for publication of clinical information and images.

## Authorship

All authors attest that they meet the current ICMJE criteria for Authorship.

## Funding

No funding or grant support.

## Declaration of competing interest

The authors declare that they have no known competing financial interests or personal relationships that could have appeared to influence the work reported in this paper.
